# A Supernumerary Soft Robotic Limb for Reducing Hand-Arm Vibration Syndromes Risks

**DOI:** 10.3389/frobt.2021.650613

**Published:** 2021-08-17

**Authors:** Andrea S. Ciullo, Manuel G. Catalano, Antonio Bicchi, Arash Ajoudani

**Affiliations:** ^1^ Soft Robotics for Human Cooperation and Rehabilitation, Istituto Italiano di Tecnologia, Genoa, Italy; ^2^ Bioengineering and Robotics Research Center “E. Piaggio”, University of Pisa, Pisa, Italy; ^3^ Human-Robot Interfaces and Physical Interaction, Istituto Italiano di Tecnologia, Genoa, Italy

**Keywords:** supernumerary robotic limbs, ergonomics, vibration suppression, assistive robotic, robotic protective equipment

## Abstract

The most common causes of the risk of work-related musculoskeletal disorders (WMSD) have been identified as joint overloading, bad postures, and vibrations. In the last two decades, various solutions ranging from human-robot collaborative systems to robotic exoskeletons have been proposed to mitigate them. More recently, a new approach has been proposed with a high potential in this direction: the supernumerary robotic limbs SRLs are additional robotic body parts (e.g., fingers, legs, and arms) that can be worn by the workers, augmenting their natural ability and reducing the risks of injuries. These systems are generally proposed in the literature for their potentiality of augmenting the user’s ability, but here we would like to explore this kind of technology as a new generation of (personal) protective equipment. A supernumerary robotic upper limb, for example, allows for indirectly interacting with hazardous objects like chemical products or vibrating tools. In particular, in this work, we present a supernumerary robotic limbs system to reduce the vibration transmitted along the arms and minimize the load on the upper limb joints. For this purpose, an off-the-shelf wearable gravity compensation system is integrated with a soft robotic hand and a custom damping wrist, designed starting from theoretical considerations on a mass-spring-damper model. The real efficacy of the system was experimentally tested within a simulated industrial work environment, where seven subjects performed a drilling task on two different materials. Experimental analysis was conducted according to the ISO-5349. Results showed a reduction from 40 to 60% of vibration transmission with respect to the traditional hand drilling using the presented SRL system without compromising the time performance.

## 1 Introduction

Work-related musculoskeletal disorders (WMSDs) are multifactorial diseases experienced by industrial workers. As discussed in da Costa and Vieira (2010), several causes can be associated with these kinds of pathologies. The most common are the heaviness of workload (Hansson et al., 2010), bad postures (Lorenzini et al., 2018), and vibration transmission (Griffin, 1997). The Italian *National Institute for Insurance against Workplace Accidents and Occupational Disease* (Istituto Nazionale Assicurazione Infortuni sul Lavoro, INAIL) reported that in 2017, in Italy, almost 53,000 statements for work-related diseases have been notified, and 65% of them were musculoskeletal related. This number increases accordingly if we enlarge the view to the whole European Union: 44 million workers showed work-related disabilities, health problems, and musculoskeletal disorders, which affect their life quality and work performance, eventually contributing to significant economic losses (Stephen et al., 2009). These issues have motivated national institutions and industries to invest resources in related research activities, with a specific focus on improving ergonomics for industrial environments.

In the robotic field, different solutions have been proposed for addressing some of the major causes of WMSDs. Joint loading reduction, for example, is one of the main goals of industrial exoskeletons (De Looze et al., 2016) and, more recently, adaptive collaborative robots (Kim et al., 2019). The use of exoskeletons, in particular, has shown significant benefits in reducing the stress on the spinal cord during heavy loading tasks (Toxiri et al. (2015). In the case of lower limbs, exoskeletons can augment the individuals’ load capacity by reducing the leg joint stress, as demonstrated in Kim et al. (2014). In contrast, for the upper limbs, shoulder and elbow joints are considered the most critical in loading tasks. In this direction, Martinez et al. presented a 5-degree-of-freedom (DOF) exoskeleton for upper limb power amplification in work environments (Martinez et al., 2008). Numerous prototypes of exoskeletons have also reached the market phase, as reported on the *“Exoskeleton Report”* website (www.exoskeletonreport.com).

Despite the high potential of the exoskeletons in improving working conditions for their wearers, they suffer from some disadvantages. One is the problem of alignment between the exoskeleton and the human joints. This aspect is very important since an exoskeleton could demonstrate an undesired effect, over-stressing the wearer’s joints and discouraging its use. Another issue, specifically related to most of the upper limb exoskeletons, is associated with the grasping action. In these devices, this action is performed by the wearer’s hands, and as a consequence, only the shoulders and/or elbow joints are supported by the device, leaving still the wrist-hand-finger joints under load. This is of particular concern when working with vibrating tools. In this case, the tool vibration is directly transmitted through the user’s hand (and accordingly to the arm), increasing the likelihood of the hand-arm vibration syndrome (HAVS). This syndrome consists of a pathological condition induced by the vibration transmitted through the hand-arm, which can affect the vascular, neurological, and/or musculoskeletal system (Aström et al., 2006). In order to deal with these kinds of disorders, different vibration features must be evaluated. The wave transmission along the arm depends on many factors, such as vibration frequency and magnitude, arm configuration (posture), and hand grasping and task forces (Griffin, 1997). Numerous studies have been conducted in this direction, analyzing how the power vibration is absorbed across the arm (Welcome et al., 2015; Dong et al., 2008). For a better understanding of this phenomenon, different models of both the human arm and the working tools have been designed and experimentally validated (Rakheja et al., 2002; Matthiesen et al., 2018).

Due to the significant relevance of this problem to the workers’ health, national (e.g., INAIL in Italy) and international (*World Health Organization*, WHO) organizations are focusing their efforts on solving it. In the European Union, a specific directive has been proposed (2002/44/EC) in order to apply a set of requirements for workers’ protection. In addition, considering the numerous factors which influence vibration transmissibility, specific international standards have been established by the *International Organization for Standardization* (see ISO-5349, −8,041, and −8,662). International directive suggests some solutions for reducing risks of HAVS, such as assuming specific postures or limiting the exposure time. Personal protective equipment has also been proposed, like anti-vibration gloves.

As discussed before, different solutions for both the load and the vibration problems have been proposed. However, to the best of our knowledge, none of them deal with both problems simultaneously. On the other hand, an interesting approach is emerging recently in the robotics field, with the potential to solve both these problems: the supernumerary robotic limbs (SRL). The SRLs are additional robotic limbs, like legs (Parietti et al., 2015), fingers (Hammond et al., 2018), or arms (Davenport et al., 2012), “worn” by the user. This new generation of robotic systems has been originally proposed to augment user ability by introducing extra limbs within the body schema. A supernumerary robotic arm-hand, for example, can help simultaneously hold and assemble aircraft fuselage structures (Parietti and Asada, 2014). Instead, the use of extra fingers allows for enhancing human hand capabilities (Prattichizzo et al., 2014; Hu et al., 2017), and additional legs can help augment balance (Parietti et al., 2015). However, the advantages of such systems are not only limited to augmenting human functions. They can significantly contribute to improving safety and ergonomics. This is the case of supernumerary robotic upper limbs used for effectively relocating loads on the wearer’s joints. Moreover, with additional artificial hands, we can also avoid directly grasping a tool, leaving the user’s hands free from direct risk exposure, like vibrations and heat.

While the limb augmentation introduced with these devices has been extensively tested and discussed in the literature (Saraiji et al., 2018; Véronneau et al., 2020; Davenport et al., 2012), no analysis has been proposed about the improvement of safety and ergonomic. With this work, we will conduct an in-depth investigation on these aspects by proposing a supernumerary robotic arm-hands system for suppressing vibration during drilling tasks and relocating the load from arm joints to shoulder and torso. A preliminary introduction of this idea was proposed in Ciullo et al. (2018), where the supernumerary robotic hand-arms system was presented. The system integrated the commercial passive steady-cam Armor Man 2.0 (Tilta Technology Co., Ltd.) with a robotic end effector, the Pisa/IIT SoftHand (Catalano et al., 2014).

Different prototypes of supernumerary robotic upper limbs have already been presented in the literature. They are generally multi-actuated, permitting the user to move and orient the additional arm freely within its workspace (Saraiji et al., 2018; Véronneau et al., 2020; Davenport et al., 2012). Although this solution allows for higher controllability of the robotic hand-arm system, identifying independent and voluntary commands for a human-supernumerary limb interface is a non-trivial issue. This can be overcome with the implementation of an autonomous control strategy, but it will increase the system complexity needing more sensors (Véronneau et al., 2020), reducing the versatility only to the implemented routines (Vatsal and Hoffman, 2018). Another alternative solution is the use of voluntary signals not involved in the task execution. For example, Saraiji et al. (2018) have proposed controlling the additional robotic arms by detecting foot movements in sitting tasks. In this case, the user naturally does not need his/her feet to perform the task so they can use them as a command generator for the control architecture; however, this limits the application scenarios. The remote control is another option for solving this issue (Véronneau et al., 2020; Vatsal and Hoffman, 2018), but this requires an additional operator to help to perform the task. Another limitation of active solutions is the need for actuation units and power sources (Véronneau et al., 2020; Nguyen et al., 2019), which increase the overall weight and encumbrance of the system, limiting its payload or discouraging its use. This can be solved by grounding the heavy parts and reducing the actuation unit dimensions. However, these solutions will impact the wearability of the system or reduce the system payload, further limiting their wide application potential. On the other hand, actuators can be specifically designed to react to the incoming vibrations and dissipate them, but the actual hardware limits on the communication bandwidth make the design more complex. For all these reasons, we have decided to adopt a passive solution for the robotic arm, integrated with one degree of actuation of the soft robotic hand. This may break the concept of “supernumerary,” since operators are requested to use their limbs to move the additional ones. However, it overcomes any control problem, still enabling users to hold multiple or large objects, relocate object/tool load on their joints, and indirectly interact with hazardous objects. In this regard, a custom passive damping wrist has been designed for connecting the robotic hand to the mechanical arm, with the aim of dissipating vibrations coming from vibrating tools. A passive implementation has been preferred over active and semi-active ones since it does not need energy sources, and it allows for a small and lightweight design, which are important criteria for wearable robotic systems. A more detailed discussion and comparison with alternative approaches will be provided in the next section.

This work provides a systematic analysis, design, and validation of the proposed system. First, a mass-spring-damper model of the arm and the robotic system is presented and used for theoretical considerations. Next, the system efficacy in vibration reduction is demonstrated through an experimental validation over seven subjects, complying with the ISO guidelines. Two different configurations are explored and compared with the natural hand condition in two different experimental scenarios, i.e., drilling SIPOREX and wood.

The structure of this paper is the following. In Section 2, functional requirements for the proposed supernumerary robotic limbs are defined. In Section 3, the system is discussed through a lumped model. In addition, a custom damping wrist is designed and implemented. The supernumerary system is described in Section 4. The experimental protocol and setup are presented in Section 5. In Section 6, the experimental results are reported and finally, in Section 7, we discuss criticism of this study and future works. Section 8 concludes this article.

## 2 Functional Requirements

As anticipated in the previous section, the proposed system is intended to reduce vibration transmission along the user’s arm and relocate loads on his/her joints during work. The typical scenario of these tasks generally implies the worker operating for many hours continuously in unstructured environments. For these reasons, the robotic system shall meet some functional and ergonomics requirements to guarantee safe and comfortable use (Howard et al., 2020). Weight and encumbrance play important roles in the overall design since a too heavy and/or too bulky architecture may discourage its usage. The use of a passive architecture for the mechanical arm provides a good trade-off between these criteria and good performance for the system goals. In particular, considering an average load compensation target in working scenarios of 7−8 kg (Altenburger et al., 2016), the spring-actuated parallelogram structure of the Armor Man largely meets these specifications providing a satisfying compensation level (see Section 4.1). This permits minimizing the active units only to the robotic hand and reducing the weight of power sources. An additional requirement is robustness to unexpected events and accidental impacts to guarantee safety during usage. In this regard, the selection of a soft robotic hand provides high robustness in accidental impacts during grasping and interaction. Another important aspect of the proposed system is related to the reduction of the vibration transmitted along the arm. To do this, we must integrate a damping element with a working frequency range of 5−25 Hz, which is assumed to be the critical frequency range in causing hand injuries, as reported in ISO 5349. In addition, this damping element must follow the same considerations of encumbrance, weight, energy consumption, and robustness. Therefore, we considered the passive solution a better option with respect to active and semi-active alternatives. Active systems are generally composed of actuator(s), sensor(s), electronic board(s), and a source of power. This design permits online monitoring of the system condition and properly reacts with the maximum efficacy (Paulitsch et al., 2004; Lew and Moon, 2001). However, due to technological limits (e.g., communication bandwidth and computational power of the control hardware), the system may become unstable. Additionally, active dampers result in increased bulky and heavy and require large energy consumption for both the actuator(s) and the electronic(s). For these reasons, this solution is generally implemented in actuated systems, where the primary role of motors regards operating and moving mechanical parts (Song et al., 2017). In this way, the damping behavior is replicated by controlling such motors adding few sensors. Nevertheless, some of the issues reported above can be overcome using semi-active dampers (Lew and Moon, 2001; Laffranchi et al., 2012). In this case, the damping effect is passively provided by the physical elements, the mechanical properties of which are properly “tuned” by the actuator(s), which allows avoiding unstable conditions. However, it requests additional elements, increasing the encumbrance and the weight of the final design. Although passive dampers do exert maximal efficacy only in a short range of conditions (compared to active and semi-active versions), they ensure a minimal impact in terms of weight, encumbrance, and energy consumption.

## 3 Theoretical Considerations

**FIGURE 1:**
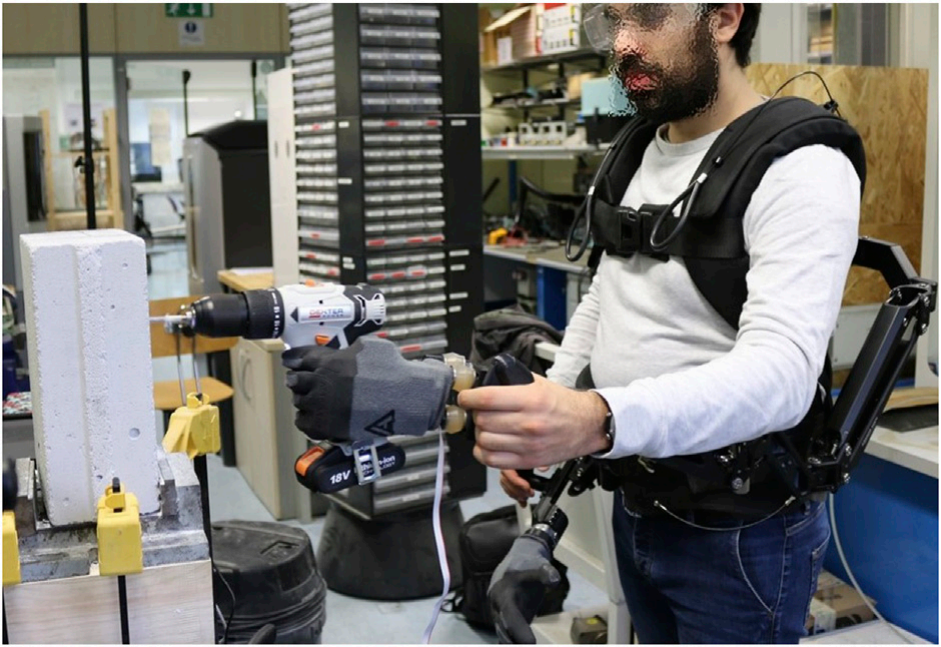
A subject drills a block of SIPOREX using the supernumerary system, which is composed of the Armor Man^®^, the Pisa/IIT SoftHand, and a customized damping wrist. The tool is held by the system, which contributes to a simultaneous load relocation and vibration transmission reduction.

With the aim of reducing muscular fatigue and vibration transmitted along the arm during work activities, a supernumerary robotic arm system was designed (see Figure 1). It was composed of two gravity compensatory arms (the Armor Man 2.0, Tilta), integrated with two robotic hands, the Pisa/IIT SoftHand (Catalano et al., 2014). The presence of the additional robotic hands allows the user to avoid grasping tools or objects directly. In this way, it is possible to relocate the load on the mechanical structures so that the operator’s joints of fingers, wrist, and elbow are not overstressed by the weight. In addition, this permits indirectly interacting with hazardous materials and objects, drastically reducing the risk of injuries. The robotic hand was connected to the compensatory arm through a custom damping wrist, specifically designed to reduce the vibration transmission along the user’s arm. A deeper technical description of the system is reported in Section 4, whereas here, some theoretical considerations are discussed.

**FIGURE 2:**
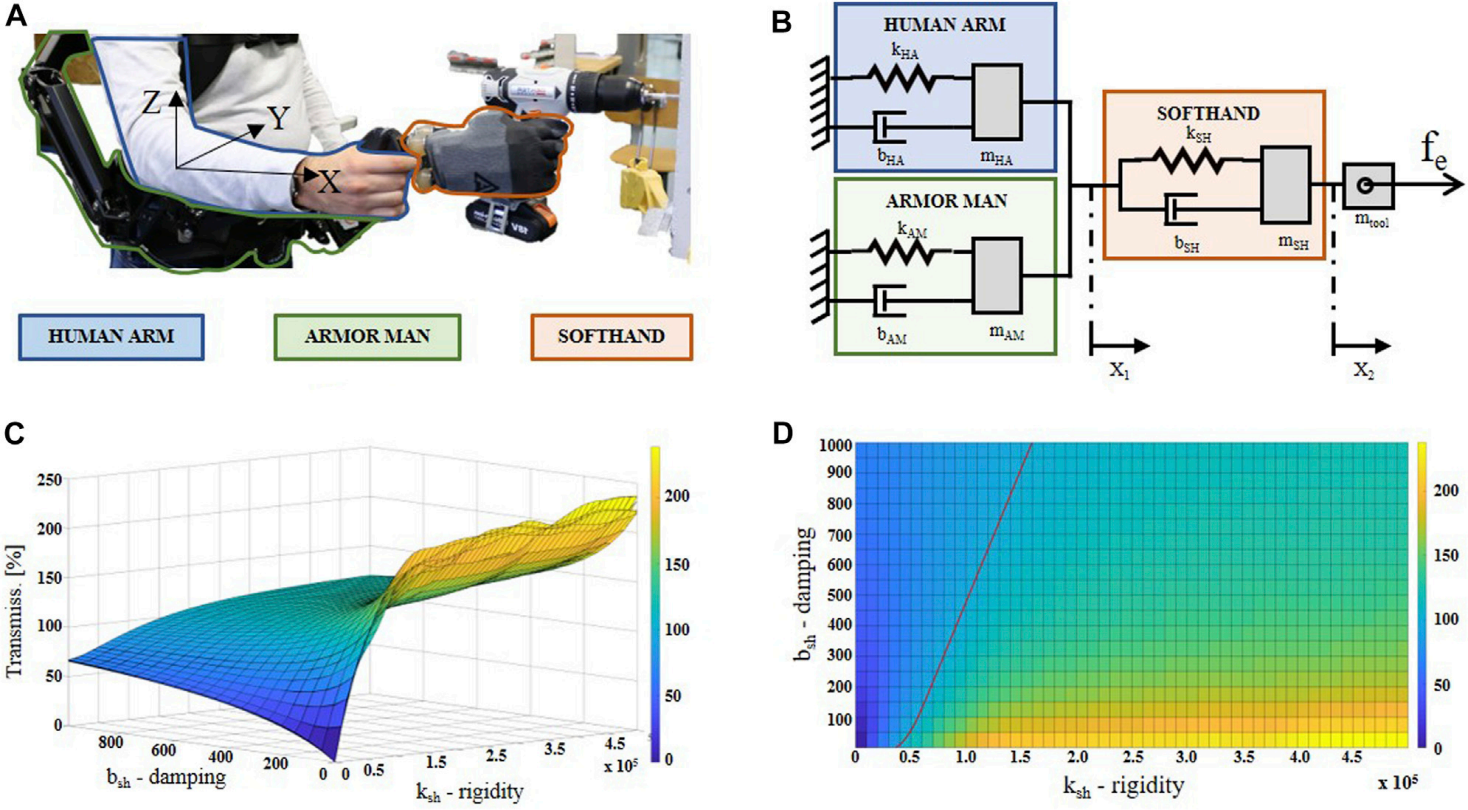
In **(A)**, the lumped model of the presented supernumerary robotic system is shown. Parameters $k_{i}$, $b_{i}$, and $m_{i}$ are, respectively, the spring stiffness, the damping, and the mass of the Human Arm (i=HA), Armor Man (i=AM), and SoftHand (i=SH) sub-models. $m_{tool}$ is the mass of the vibrating working tool that imposes an external force $f_{e}$. In **(B)**, the three sub-models are highlighted with colors on a subject wearing the system and holding a drill. In **(C)** and in **(D)**, the vibration transmissibility from $m_{SH}$ to $m_{HA}$ is plotted (3D and top view, respectively), varying the $k_{SH}$ and $b_{SH}$ values. The red line in **(D)** represents the 100% transmissibility border: on the right of the line, the transmissibility is over 100%.

While the joint load relocation provided by this SRL system could appear evident, vibration reduction requires a deeper analysis. Therefore, a simplistic lumped model of the system was explored with a one-direction analysis. In particular, during the drilling task, the drill bit is generally aligned with the user’s arm. For this work, this direction was considered the most affected direction for vibration transmission. In the following, we will refer to this as *x*-direction (or arm direction), according to a forearm-fixed reference frame with the *x*-axis parallel to it, pointing toward the hand (see Figure 2A). The system, integrated with the user’s arm, can be represented by a 2-DOF mass-spring-damper model, as shown in Figure 2B. The model was composed of three main sub-models, which represented, respectively, the human arm (HA), the gravity compensatory arm (Armor Man, AM), and the robotic hand with the damping wrist (SoftHand, SH). When a user grabs the distal part of the gravity compensatory arm (as shown in Figure 2A), the HA can be considered connected in parallel with the AM, and together they are serially connected to the SH through the damping wrist, which is represented by its physical properties of rigidity ($k_{SH}$) and damping ($b_{SH}$). In the same way, the other sub-models are represented with their own parameters, i.e., the inertial masses ($m_{HA}$, $m_{AM}$ and $m_{SH}$), rigidity ($k_{HA}$ and $k_{AM}$), and damping ($b_{HA}$ and $b_{AM}$). An additional mass ($m_{tool}$), rigidly connected to $m_{SH}$, represented the mass of a hypothetical vibrating working tool. Although directly referring to the damping wrist, for simplicity of modeling, $k_{SH}$ and $b_{SH}$ also include the physical properties of the robotic hand.

It is worth noting that it is out of the scope of this work to design and estimate a new human arm model, and this section does not aim to provide an in-depth model identification analysis. With the following analysis, we are mainly oriented to theoretically contextualize and define the proposed system into the highlighted problem. For this reason, the HA sub-model is a 1-DOF mass-spring-damper model inspired by (Reynolds and Soedel, 1972), whose parameters are reported in the ISO 10068:2012 document. It should also be noted that the model used here is grounded, where the ground is represented by the rest of the human body over the shoulder level. This is in line with the assumption generally proposed in the literature for dealing with this kind of analysis (Rakheja et al., 2002; Matthiesen et al., 2018; Dong et al., 2007; Dong et al., 2013). Similar to the state of the art, in this work, the involved vibration magnitude is low enough for neglecting its transmission on superior substructures as the head, torso, and legs. Regarding the AM sub-model, the main influence in terms of damping and rigidity is caused by the padding of the vest, where the gravity compensatory arms are attached. This is because its vertical joints make the arm totally free to move in the investigated direction. The dynamic parameters $k_{AM}$ and $b_{AM}$ for this sub-model were inspired by White et al. (2000), and $m_{AM}$ was measured.

The dynamic response in the *x*-direction of the entire model is described by the following equations:

$$\{\begin{array}{cc} \left(m_{SH}+m_{tool}\right){\ddot{x}}_{2}+b_{SH}\text{Δ}\dot{x}+k_{SH}\text{Δ}x=f_{e} & \\ m_{eq}{\ddot{x}}_{1}+b_{eq}{\dot{x}}_{1}+k_{eq}x_{1}=b_{SH}\text{Δ}\dot{x}+k_{SH}\text{Δ}x & \end{array},$$ (1)

where $m_{eq}$, $b_{eq}$, and $k_{eq}$ are, respectively, the sum of the parameters of the sub-models AM and HA, $x_{1}$ and $x_{2}$ are the hand and the drill mass point along the *x*-direction, respectively (as shown in Figure 2B), $\text{Δ}\dot{x}=\dot{x_{2}}-\dot{x_{1}}$, $\text{Δ}x=x_{2}-x_{1}$, and $f_{e}$ is the external force. In order to simulate the vibration on the human hand (${\ddot{z}}_{1}$), this equation system was implemented on Simulink 2019a. For this purpose, the acceleration on $m_{tool}$ during a real drilling task (i.e., the time series of ${\ddot{x}}_{2}$) was recorded and used as input into the model (more details on the experimental setup in Section 5). In particular, the vibrations generated by drilling into a piece of wood were used for these simulations. For this analysis, we focused on the effects provided by the custom damping wrist by simulating the system with different values of $k_{SH}$ and $b_{SH}$.

Considering the EU directive (2002/44/EC) and the ISO 5349, the vibration effect from a working tool must be evaluated by observing the acceleration transmitted on the human arm. In particular, the daily exposure A(8), i.e., the level of vibration exposure over an 8 h work period, needs to be estimated. It is calculated as follows:

$$A\left(8\right)=a_{hv}\sqrt{\frac{T_{e}}{T_{0}}},$$ (2)

where $a_{hv}$ is the total vibration value of frequency-weighted root mean square (r.m.s.) acceleration during the exposure, $T_{e}$ is the total duration of the exposure during one workday, and $T_{0}$ is the reference duration of 8 h. As reported in Eq. 2, for evaluating the A(8), $a_{hv}$ needs to be measured. According to the ISO 5349, $a_{hv}$ corresponds to “*the root-sum-of-squares of the* $a_{hwi}$ *values for the three measured axes of vibration, in meters per second squared* ($m/s^{2}$)*”*:

$$a_{hv}=\sqrt{a_{hwx}^{2}+a_{hwy}^{2}+a_{hwz}^{2}},$$ (3)

where $a_{hwi}$ is the r.m.s. frequency-weighted acceleration on the *i*-axis. This is obtained by filtering the raw acceleration, measured on each axis, with the frequency-weighting and band-limiting filter (later ISO filter) defined in the ISO documentation and then performing the root mean square. Next, to estimate the efficacy of the custom damping wrist with the presented model, we calculated the transmissibility as the ratio of the acceleration on the human hand over the acceleration on the vibrating tool, both filtered with the ISO filter. Following the notation used on the model, the transmissibility was calculated as follows:

$$T\;\left[\text{\%}\right]=\frac{a_{1hv}}{a_{2hv}}\cdot 100,$$ (4)

where $a_{1hv}$ and $a_{2hv}$ are the total vibration value of frequency-weighted r.m.s acceleration of $m_{HA}$ and $m_{tool}$, respectively.

As shown in Figures 2C,D, simulation results demonstrate how the custom wrist rigidity and damping parameters act on the vibration transmissibility. The first consideration comes from null values for both the parameters: as expected, this condition results in 0% vibration transmission. This is due to the fact that null values of $k_{SH}$ and $b_{SH}$ correspond to uncoupling the SH sub-model from HA and AM. In this way, any vibration provided on $m_{SH}$ can not be propagated toward $m_{HA}$. Unfortunately, this condition is unfeasible since any kind of physical connection is associated with a certain value of rigidity ($k_{SH}$) and damping ($b_{SH}$).

Different transmissibility trends can be observed by augmenting one of the two parameters per time. Supposing a fixed $b_{SH}$ at 0 and augmenting $k_{SH}$ progressively, the vibration starts to propagate more and more until the wrist acts as a vibration amplifier, i.e., T>100% (see the $k_{SH}$ axis of Figure 2D, for values on the right of the red line). Instead, if we fix $k_{SH}$ at 0 and augment $b_{SH}$ progressively, vibration will still be transmitted from $m_{SH}$ to $m_{HA}$, but with less energy. In fact, the transmissibility never goes over 100%, since the dampers dissipate most of the vibration (see the $b_{SH}$ axis of Figure 2D). Although a pure damping wrist would be the best solution in terms of vibration reduction, it would be inefficient in terms of joint load reduction. In fact, due to the low rigidity, the robotic hand will bend downwards as soon as a load will be applied on it. This effect will compromise one of the two main benefits of this SRL system, i.e., the load relocation on the user’s joints. For this reason, a good trade-off can be medium/low rigidity and high damping effect in order to offer benefits for both vibration and load issues. Other important constraints arise from the physical dimension of the system and the frequency spectrum of vibration. The first suggests small and light components for the wrist; otherwise, the resulting system will be heavy and bulky, discouraging its use. The second, instead, requires to dissipate as much as possible the vibration within the 5−25 Hz range, which is assumed to be the critical frequency range in causing hand injuries, as reported in ISO 5349.

**FIGURE 3:**
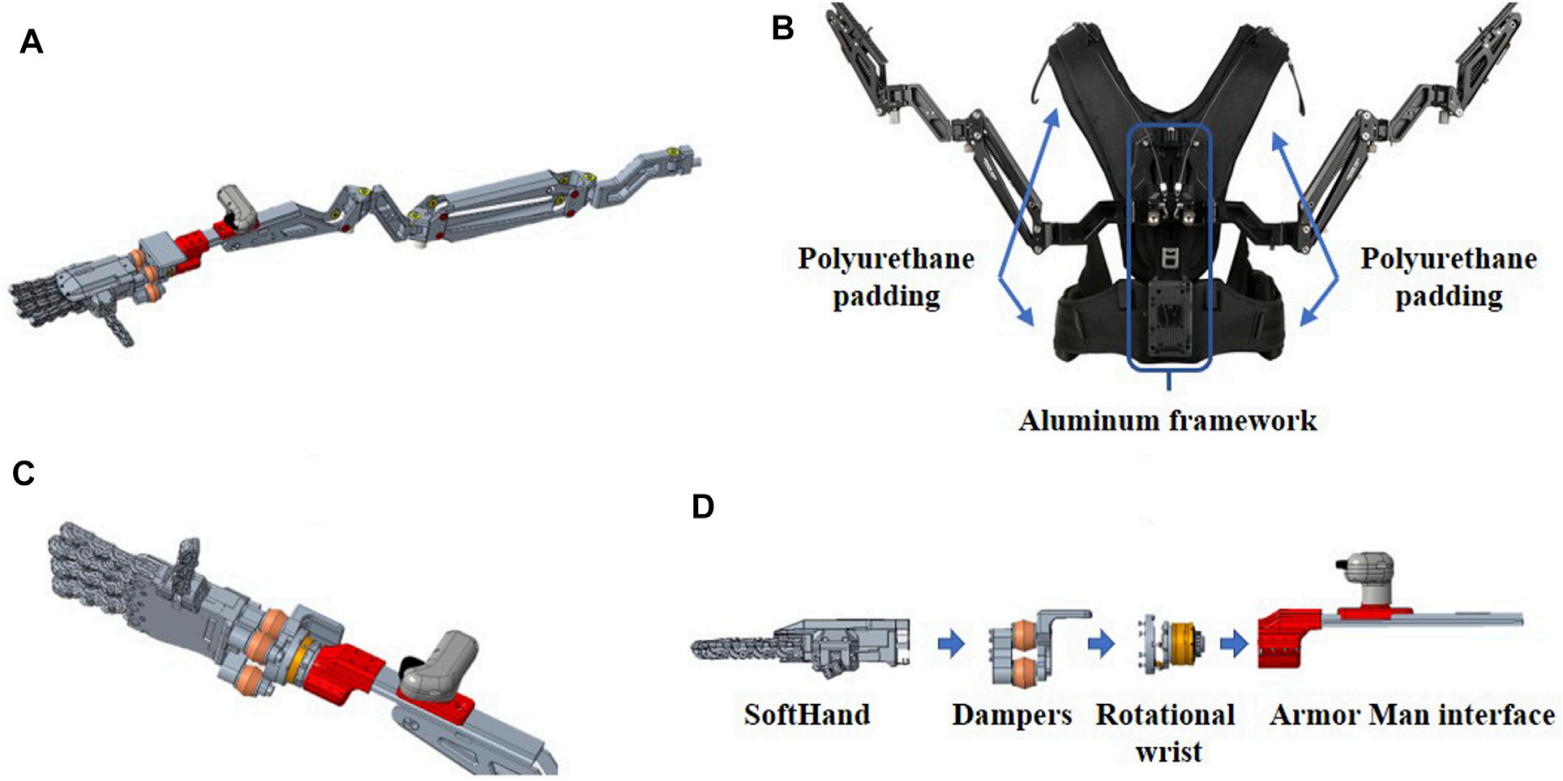
The 3D CAD model of the SRL is shown. Picture **(A)**: the whole compensatory arm integrated with the robotic hand through the custom damping wrist. Picture **(B)** shows the Armor Man, highlighting main parts. Picture **(C)**: details of the robotic hand with the custom damping wrist and the user handle. Picture **(D)**: the exploded view of the hand-wrist assembly.

According to the simulation results and the system requirements listed above, the most optimal solution needs to have small (few centimeters) and light (less than 0.5 kg) dampers acting in the low-frequency spectrum (5−25 Hz) and low-medium rigidity (less than 1 MNs/m). These strict requirements drastically narrow the range of useful dampers commercially available. After a meticulous survey among the suitable commercial solutions and preliminary tests with a few selections of them, the gel-based MN-5 (MISUMI) dampers were chosen. In particular, a parallel assembly of four of them was serially mounted between the compensatory arm and the robotic hand, as shown in Figures 2C, 3B. They comply with the size (2.2 cm long, 3 cm diameter), weight (30 g per damper), load capacity (up to 200 N), and rigidity (ca. 21 kNs/m per damper) criteria. The dampers’ effective frequency is from 16 Hz, based on the datasheet information, which is the middle of the target frequency range.

## 4 System Description

As mentioned in the previous sections, the new supernumerary robotic arm-hand system is composed of two gravity compensatory arms (the Armor Man 2.0, Tilta), integrated with two robotic hands, the SoftHand (Catalano et al., 2014), through a customized wrist damping mechanism. The use of additional robotic hands as end effector makes the system multi-purpose oriented since it allows for grasping many different kinds of objects and tools, varying in size and shape. This is an important feature both for expanding the user’s grasping capacity, like multiple objects/tools grasping or holding large objects, and for reducing hand risk exposure to hazardous materials or tools.

### 4.1 Gravity Support

The gravity compensatory arms (2 kg each, shown in Figure 3A) are fixed on a wearable vest (3 kg), composed of a central aluminum plate and covered with polyurethane padding (see Figure 3B). The gravity compensatory action on each arm is performed thanks to two springs mounted on a parallelogram and a triangular structure, respectively. Their reaction forces counteract the weight of the attached damping wrist, the robotic hand, and the grasped object or tool, if any, up to 22 kg (11 kg each arm). In this way, the user only needs to “drive” the system for approaching the object/tool with the robotic hand, with very little muscle effort.

### 4.2 Robotic Hand and Custom Damping Wrist

In order to grasp and manipulate objects without overloading the user’s wrist-hand-finger joints, an under-actuated and synergy-driven robotic hand (0.7 kg) is mounted as an end effector on the compensatory arms (one per arm) as shown in Figure 3. The specific finger joint mechanism of the hand allows different grasps by commanding the same closure pose. The robotic fingers can adapt to the object’s shape during the grasping action, ensuring a reliable grasp. The hand closure is controlled by using a custom handle with a lever that proportionally sets the closure pose of the hand. This handle is also used by the user to steer the robotic arm to direct the softhand toward the target. The hand mechanism is powered by a Maxon Motor DCX22S equipped with a GPX22 planetary gearbox. The low-level control is implemented on a custom electronic board (based on Cypress Programmable System on Chip-PSoC, with RS485 communication protocol), as discussed in (Santina et al. (2017)). The connection between the robotic hand and the gravity compensatory arm is made through the custom damping wrist (0.3 kg), as shown in Figure 3C. The wrist is composed of two functional parts, i.e., the rotational joint and the damping elements (see Figures 3B,C). The first is implemented by an aluminum prosthetic-like wrist, which permits the pro-supination rotation of the robotic hand. The second is implemented by the four MN-5 dampers (see Section 3), which dissipate the vibration coming from the vibrating tool grasped by the robotic hand.

## 5 Experimental Analysis

For investigating and quantifying the vibration reduction provided by this system, a work activity was simulated experimentally. Hand vibrations of seven male subjects were measured during a drilling task. Two different materials, SIPOREX and wood, were drilled with two different drill configurations. In this way, the system performance can be validated against different conditions. The two materials differ in terms of density, hardness, and friability, so there would be different vibration absorption, thus varying the quantity of vibration transmitted to the subject. In addition, the two drill configurations can introduce different kinds of vibrations in terms of magnitude, frequency, and directions.

### 5.1 Experimental Setup

**FIGURE 4:**
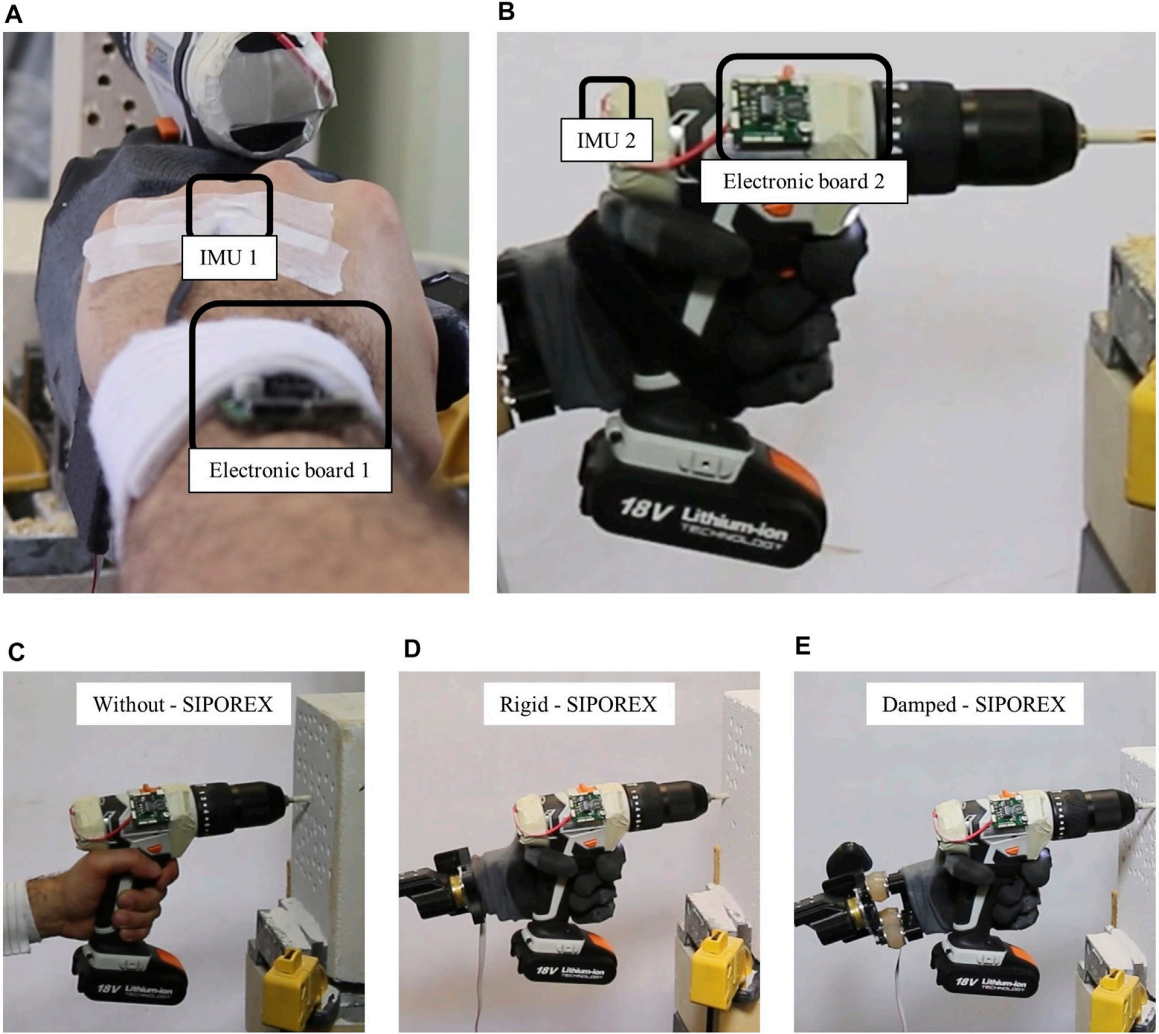
**(A,B)** The IMUs and the electronic boards for recording the acceleration on both the hand and the drill. **(C–E)** The *drilling in* phase with the SIPOREX, for the *without*, *rigid*, and *damped* configurations, respectively.

To measure the vibration levels, two Inertial Measurement Units (IMUs) MPU-9250 MotionTracking (by InvenSens, Inc., San Jose, CA, United States) were used: one fixed on the dorsal side of each subject’s hand (see Figure 4A) and one on the backside of the drill (see Figure 4B). The acceleration from both IMUs was acquired by two custom electronic boards (Santina et al., 2017) (based on Cypress Programmable System on Chip-PSoC, with RS485 communication protocol), as shown in Figures 4A,B, with a sample rate of 1 kHz. The electronic boards were connected to a PC laptop (Dell Inspiron 15 7,559 Gaming Series; CPU: Intel i7 6,700 at 2.60 GHz; RAM: 16 GB; OS: Windows 10–64 bit) by USB cables and remotely managed with a custom C++ interface. The acceleration from both IMUs was recorded simultaneously, together with the CPU time clock, in order to guarantee the synchronization of data. The drilling task was conducted using a DEXTER CDI219LD hammer drill (Li-Ion battery, 18V-2Ah; Max Torque: 40 Nm; No-Load speed: 400–1,400 rpm; Impact Rate: 6,400–24,000 blows-per-minute).

### 5.2 Experimental Protocol

To evaluate each system component's contribution (i.e., the robotic hand and the damping wrist) to the vibration reduction, a drilling task experiment was designed as follows. Seven male subjects were asked to make 12 holes in three different configurations:

• W*ithout*: by holding the hammer drill with their own hand (see Figure 4C);
• Rigid: by holding the hammer drill with the robotic hand rigidly connected to the compensatory arm with four screws (see Figure 4D);
• Damped: by holding the hammer drill with the robotic hand connected to the compensatory arm with the four dampers (see Figure 4E).

Each subject was asked to drill, with each configuration (see Figures 4C–E), both a block of SIPOREX and a piece of wood. To do this, the hammer drill was set at the maximum speed (1,400 rpm) impact/hammer OFF for the wood and at the minimum speed (400 rpm), and impact/hammer ON (6,400 bpm) for SIPOREX. The drilling task protocol, composed of four different phases, was fixed as follows:

1) *Drill off*: holding the drill while it is OFF;
2) *Drill on*: holding the drill after turning it ON;
3) *Drilling in*: drilling the material;
4) *Drilling out*: removing the drill from the material.

The identification of four phases for the drilling action was necessary because of the differences in time duration for each of them. According to the ISO 5349 document, the vibration evaluation is based on the *r.m.s.* values of the frequency-weighted acceleration vectors on each axis. However, during a hole drilling performance, stronger vibrations are generated during the drill bit interaction with the material, which generally is the fastest phase. On the contrary, a larger time is spent holding the drill and positioning it. In this way, calculating the *r.m.s.* on the entire action, the non-interacting phase may induce an underestimation of the real vibration exerted during the task.

**FIGURE 5:**
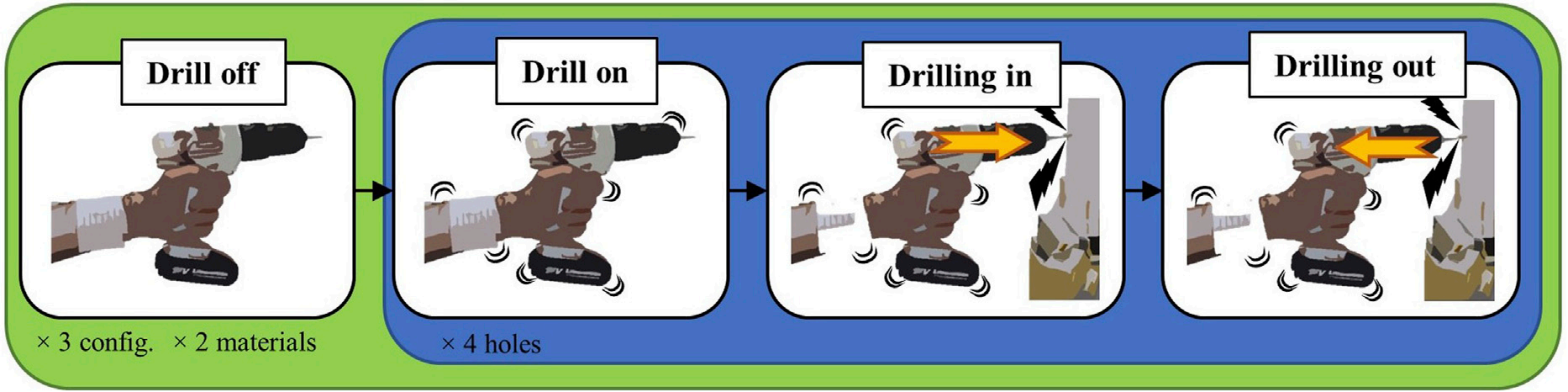
Workflow of the different experimental phases.

Some precautions were considered to avoid differences in vibration transmission due to the robotic hand grasping force and drill velocity rotation. First, the drill trigger was maximally pushed using a Velcro strap. Then, the drill was fixed at the robotic hand, which grasped it with the maximum force at the beginning of the experiment and released it at the end. To stop the drilling, the battery connection/disconnection was used as an ON/OFF system. This was necessary between phases 1 and 2, where the subject needed to turn on the drill. Then, for the *drilling on* phase, each subject was asked to insert the drill bit for 5 cm. To do this, a colored tape was attached at the beginning of the drill bit, leaving only 5 cm free for drilling. The subject was instructed to stop drilling in the material once the tape reached the hole. Then, the *drilling out* phase started and the subject moved the drill back. The start of phases 1, 2, and 3 was vocally announced by the experimenter. He also reported all the four starting phases on the C++ interface by pressing the bar space. The subject was asked to make four holes in a row, so the entire procedure (for one configuration and one material) was repeated three times. The subject was free to decide the hole position on the material, with the only constraint to not make more than one hole on the same point. Each block of material was fixed with a vise on a workbench, positioned around 100 cm far in front of the subject, 130 cm from the floor. Block dimensions were 30×40×10 cm for SIPOREX and 20×7×7 cm for wood. SIPOREX blocks were drilled first, starting with the *without* configuration, then *rigid* and *damped*. Once SIPOREX blocks were finished, wood blocks were fixed and drilled with the same drill configurations order. The subject was asked to try performing all the holes applying the same force. The whole workflow is shown in Figure 5.

### 5.3 Data Analysis

In order to compare the effect of the different configurations tested, a within-subject analysis was conducted directly on the $a_{hv}$ values (measured on the hand and the drill), instead of the A(8). This can be done if we consider the same amount of working time ($T_{e}$ in Eq. 2) for all the three configurations (*without*, *rigid*, and *damped*). In particular, for all the six trials (3 configurations × 2 materials), a value for each of the phases with the drill ON (i.e., phases *2*, *3*, and *4*) for all the 12 holes was measured. In this way, for each trial, 12 (holes) × 3 (phases) values were measured so that a statistical analysis can be conducted for comparing the three different configurations. Before statistical investigations, the default Matlab (version 2019a) function *rmoutliers*, with *median* option, was used for detecting and removing outliers. With this function, all values greater than three times the scaled median absolute deviations were considered outliers and removed. Then, the Lilliefors test (α=0.05) was performed to assess if data were normally distributed. The test was positive (normally distributed) for all the samples, so a parametric statistical analysis (one-way ANOVA, α=0.05) was conducted on the hand acceleration results in order to compare the three different configurations for each subject. Due to the small sample dimension (only seven subjects), no statistical analysis was conducted across subjects. All analyses were conducted with Matlab 2019a.

In addition to the $a_{hv}$ values, the transmissibility percentage ratio (*T*), which indicates the vibration transmitted from the drill to the hand, was calculated as reported in Section 3. In this case, the acceleration used corresponds to $a_{hv}$ measured on the hand and the drill, as follows:

$$T\;\left[\text{\%}\right]=\frac{a_{hv}^{hand}}{a_{hv}^{drill}}\cdot 100,$$ (5)

where $a_{hv}^{hand}$ and $a_{hv}^{drill}$ are $a_{hv}$ measured on the hand and on the drill, respectively. Then, we measured and reported the execution times for the *drilling in* and *drilling out* phase. Finally, the frequency spectrum of the hand vibration along the three axes for all the subjects was evaluated. To do this, the Discrete Fourier Transform (DFT) per each hole per each subject was computed (12×7=84 DFT) along the three axes using the default fast Fourier transform (*fft*) algorithm implemented on Matlab (version 2019a). Then, the median DFT (± the interquartile range) among the 86 DFT per each axis was calculated and plotted. These evaluations were limited only to the *drilling in* phase, which was considered the most representative and affected of larger vibrations in this task.

### 5.4 Ethics

The study was reviewed and approved by the Regional ethics committee of Liguria (Protocol IIT-HRII-ERGOLEAN, 156/2020, DB-id 10,215). The participants provided their written informed consent to participate in this study and for the publication of any potentially identifiable images or data included in this article. The data that support the findings of this study are available from the corresponding author upon request.

## 6 Results

**FIGURE 6:**
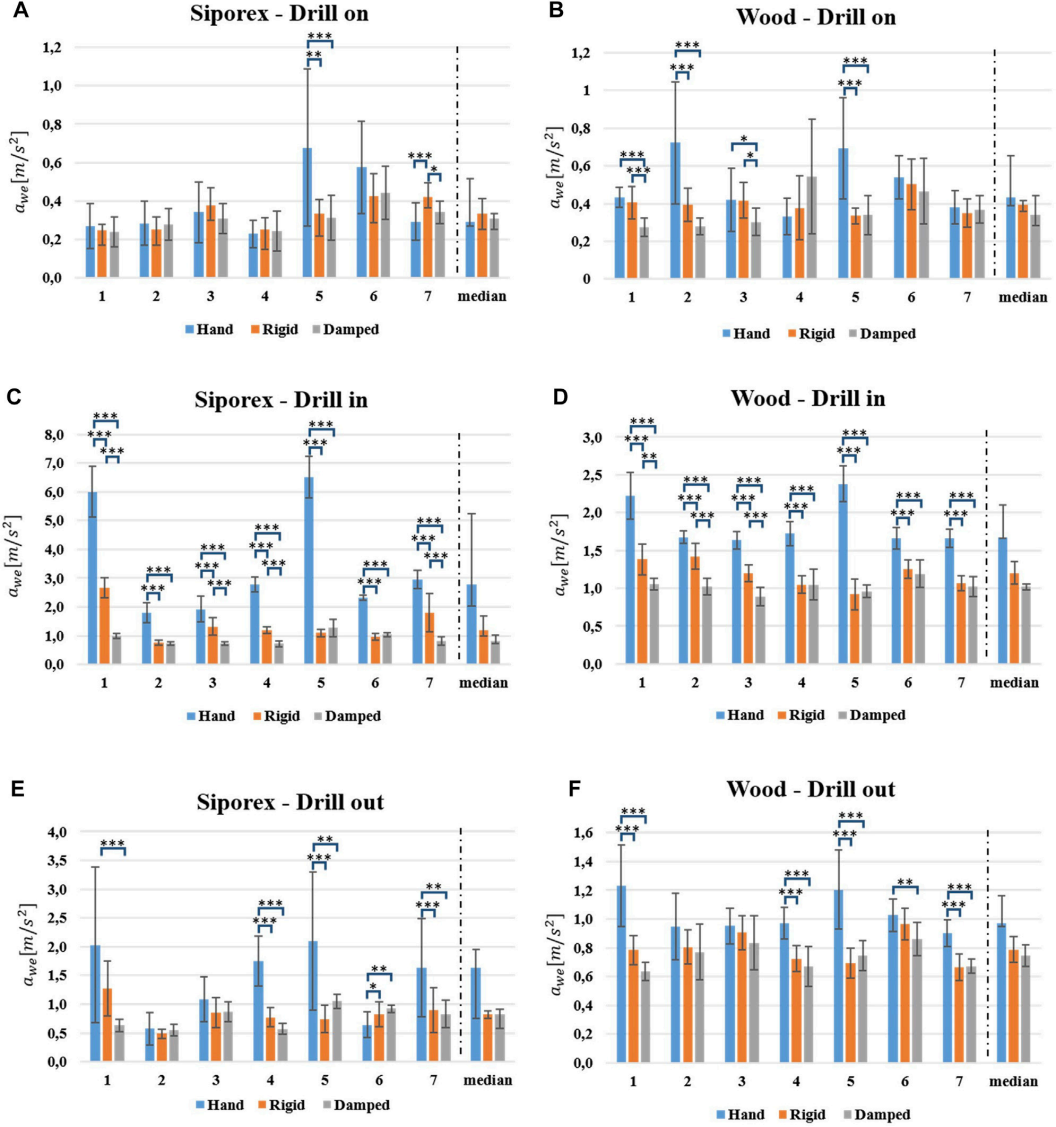
The $a_{hv}^{hand}$ for each subject and the median values among the subjects (separated by the dotted line in each plot) are reported for both the SIPOREX and the wood experiments. **(A,C,E)** Acceleration values for the SIPOREX *drill on*, *drilling in*, and *drilling out* phases. Plots **(B,D,F)** show acceleration values of the same phases for drilling wood. Statistical differences legend: * = p<0.05, ** = p<0.01, *** = p<0.001. No statistical investigation has been conducted for median values.

Numerical results are reported in Table 1 and graphically shown in Figure 6. SIPOREX results are presented first, followed by the wood results. The median values among the subjects are used for discussion. The outlier removal function identified and discarded a total of 73 outliers on the whole dataset (2,272 values).

**TABLE 1:** Experimental results.

	SIPOREX			Wood		
	W/O	R	D	W/O	R	D
Drill on					
Hand [$m/s^{2}$]	0.292	0.333	0.308	0.435	0.394	0.340
[Q1 Q3]	[0.271, 0.516]	[0.252, 0.410]	[0.253, 0.333]	[0.389, 0.655]	[0.357, 0.414]	0.440]
Drill [$m/s^{2}$]	2.873	3.180	3.204	3.807	3.654	3.480
[Q1 Q3]	[2.604, 4.554]	[2.685, 4.843]	[2.727, 3.862]	[3.619, 4.212]	[3.375, 3.944]	[3.367, 4.015]
Trans. [%]	10.2	10.5	9.6	11.4	10.8	9.8
Stat.	W/O vs. R	W/O vs. D	R vs. D	W/O vs. R	W/O vs. D	R vs. D
	2	1	1	2	4	2
Drill in						
Hand [$m/s^{2}$]	2.783	1.200	0.819	1.676	1.199	1.024
[Q1 Q3]	[2.025, 5.239]	[0.997, 1.685]	[0.721, 1.027]	[1.662, 2.102]	[1.054, 1.351]	[0.976, 1.052]
Drill [$m/s^{2}$]	15.48	17.36	13.53	5.973	6.220	6.151
[Q1 Q3]	[12.54, 18.83]	[14.76, 17.84]	[12.57, 17.74]	[5.869, 6.499]	[5.837, 6.508]	[5.706, 6.436]
Trans. [%]	18.0	6.9	6.1	28.1	19.3	16.6
Time [s]	3.26	3.22	3.38	2.34	2.09	2.33
Stat.	W/O vs. R	W/O vs. D	R vs. D	W/O vs. R	W/O vs. D	R vs. D
	7	7	4	7	7	3
Drill out						
Hand [$m/s^{2}$]	1.633	0.829	0.831	0.973	0.784	0.745
[Q1 Q3]	[0.752, 1.960]	[0.749, 0.889]	[0.586, 0.909]	[0.950, 1.160]	[0.701, 0.880]	[0.671, 0.819]
Drill [$m/s^{2}$]	8.420	5.990	5.645	5.350	5.200	5.018
[Q1 Q3]	[5.083, 8.478]	[3.979, 7.124]	[4.317, 8.182]	[4.642, 5.424]	[4.781, 5.260]	[4.818, 5.214]
Trans. [%]	19.4	13.8	14.7	18.2	15.1	14.8
Time [s]	1.03	1.25	1.18	1.27	1.26	1.29
Stat.	W/O vs. R	W/O vs. D	R vs. D	W/O vs. R	W/O vs. D	R vs. D
	4	5	0	4	5	0

In this table, experimental and statistical results of drilling SIPOREX and wood are reported in terms of hand and drill median (first - third quartile) vibration, transmissibility, mean drilling time (for *drilling in* and *drilling out* phases) among the subjects, and the number of subjects showing statistical differences. W/O, without configuration; R, rigid configuration; D, damped configuration; Trans., transmissibility; [Q1 Q3], first and third quartile.

### 6.1 Drilling SIPOREX

During the *drill on* phase, the acceleration value measured on the hand ($a_{hv}^{hand}$) and the transmissibility (*T*) were the lowest without the robotic system ($a_{hv}^{hand}$: $0.281\;m/s^{2}$; T=7.1%). Statistical differences were found for 5 subjects out of 7 comparing the *rigid* configuration against the *without*, for 4 subjects out of 7 comparing the *damped* configuration against the *without*, whereas none of the subjects showed differences between the two robotic configurations.

**FIGURE 7:**
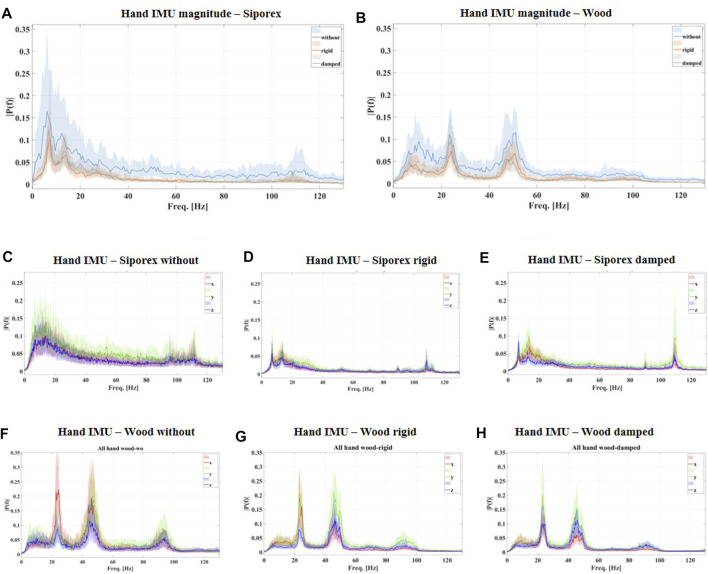
frequency spectra of the hand vibration during the *drilling in* phase for all the subjects are shown. In particular, **(A,B)** the spectrum of the magnitude acceleration for the three different configurations (*without* in light-blue, *rigid* in orange, and *damped* in gray) drilling in SIPOREX **(A)** and wood **(B)**. **(C,D,E)** The frequency spectrum of the acceleration along the three axes (x in red, y in green, and z in blue) for the three different configurations in drilling SIPOREX; **(F,G,H)** the frequency spectrum of the acceleration along the three axes (x in red, y in green, z in blue) for the three different configurations in drilling wood. All the curves represent the median Discrete Fourier Transform among all the holes of all the subjects. Color bands represent the interquartile range.

During the *drilling in* phase, both $a_{hv}^{hand}$ and *T* values were the lowest for the *damped* configuration ($0.818\;m/s^{2}$; 6.0%). Statistical differences were shown for all the subjects for both the robotic configurations vs. the *without* ones, whereas they were only observed in 4 subjects out of seven between the two robotic configurations. Execution time for this phase resulted in ca. 3 s for all the configurations (difference of just 100 ms between the robotic configurations and the *without* one). The frequency spectrum of the robotic configurations (see Figure 7A) showed an important power reduction on low frequencies (below 50 Hz) with respect to the hand drilling.

For the last phase, the *drilling out*, both $a_{hv}^{hand}$ and the *T* values were the lowest for the *rigid* configuration ($0.828\;m/s^{2}$; 13.8%). According to the statistical analysis, 3 subjects out of 7 showed statistical differences for the *rigid* configuration against the *without* one and 5 out of 7 showed statistical differences for the *damped* configuration against the *without*. In contrast, the statistical difference between the two robotic configurations was found only in 1 subject. Execution time for this phase was ca. 1 s for all the configurations (a difference of only 200 ms between the robotic configurations and the *without* one).

### 6.2 Drilling Wood

Regarding the *drill on* phase, both $a_{hv}^{hand}$ and the *T* values were the lowest for the *damped* configuration ($0.340\;m/s^{2}$ ; 9.1%). The robotic system showed statistical differences in 4 subjects out of 7 for the *rigid* configuration against the *without* and in 6 subjects out of 7 for the *damped* against the *without*, whereas differences between the two robotic configurations were found in 3 subjects out of 7.

In the *drilling in* phase, both $a_{hv}^{hand}$ and the *T* values were the lowest for the damped configuration ($1.023\;m/s^{2}$; 16.6%). Statistical analysis indicated statistical differences in all the subjects for both the *rigid* and the *damped* configuration against the *without* ones, while in only 4 subjects out of 7, differences were found between the two robotic configurations. Execution time for this phase was ca. 2.3 s for all the configurations (differences of only 200 ms between the robotic configurations and the hand). The frequency spectrum of the robotic configurations (see Figure 7B) showed a slight power reduction on low frequencies (below 50 Hz) with respect to the hand drilling.

Moreover, for the last phase, the *drilling out*, the $a_{hv}^{hand}$ and the *T* values were the lowest for the damped configuration ($0.706\;m/s^{2};13.9\text{\%}$). Statistical differences were found in 4 subjects out of 7 for the *rigid* configuration against the *without*, in 5 subjects out of 7 for the *damped* configuration against the *without*, and in only 1 subject comparing the two robotic configurations. Execution time for this phase was ca. 1.3 s for all the configurations (no differences among them).

## 7 Discussions

In this work, the hand-transmitted vibration intensity was estimated through the index defined by ISO-5349, which uses the acceleration measurements on the subjects’ hands. Numerical results show an important reduction of the vibration transmission during drilling tasks, thanks to the proposed robotic system. In particular, during the *drilling in* phase, a reduction of the vibration transmission of the 60% (from 18 to 6% for SIPOREX) and of the 40% (from 28 to 16% for wood) has been measured (see Table 1). Interestingly, the absolute vibration, measured on the subjects’ hands ($a_{hv}^{hand}$) while using the robotic system equipped with the custom damping wrist, always respected the EU directive (2002/44/EC) regulations on the “*minimum health and safety requirements regarding the exposure of workers to the risks arising from physical agents*”^1^. According to this directive, for any work activity to be considered safe, the A(8) (see Eq. 2) index needs to be under specific thresholds: $2.5\;m/s^{2}$ (*exposure action value*) and $5\;m/s^{2}$ (*exposure limit*). If the measured A(8) is within the *exposure action value* and the *exposure limit*, the employer should program and implement different solutions for reducing the worker’s exposure, e.g., by planning adequate rest periods and appropriate maintenance of work equipment or providing alternative working methods. On the contrary, if the index goes over the *exposure limit*, immediate actions must be taken to reduce it. In the case of a duration exposure ($T_{e}$ in Eq. 2) equal to the reference duration time ($T_{0}$ in Eq. 2), the A(8) coincides with $a_{hv}^{hand}$. This was considered the case limit in our experiments, i.e., 8 h of continuous drilling with the setup described in Section 5. As shown in Figure 6C, over the threshold, $a_{hv}^{hand}$ values were recorded in 4 subjects out of 7 for the SIPOREX *drilling in* phase (drilling + hammering) without the robotic system. In contrast, as reported in both Figure 6 and Table 1, none of the $a_{hv}^{hand}$ measured when using the robotic system went over the lower threshold. This is an important achievement for the safety of workers since it means that the designed SRL system can strongly reduce the risks related to the HAVS (House (2010)). Although the median values among the subjects (see Table 1 and Figure 6) showed a decreasing trend in the vibration transmission for the robotic system with respect to the hand drilling, a statistical validation was not possible due to the small sample size (7 subjects). A larger subject sample size will be included in further investigations to provide a more robust statistical evaluation. Apart from this analysis, only one tool (in two configurations) was tested. It is worth noting that the proposed system can be potentially used with many others (e.g., polishers, grinders, or jigsaws). Different from most of the solutions reported in the literature, which are generally embedded into the tool design (Hao and Ripin, 2013; Tewari and Dewangan, 2009; Ko et al., 2011), the vibration suppression architecture here proposed is highly versatile thanks to the presence of the anthropomorphic soft robotic hand.

To estimate the damping effect of the proposed SRL, the arm-hand acceleration was measured only on the back of the individuals’ hands; hence, the effects of vibration on sub-structures (e.g., palm skin and fingers) or distant ones (e.g., forearms, elbow and shoulder) were not investigated. This choice was motivated by the fact that the more distant the vibration source, the less its transmission (Xu et al., 2017). This implies that the back part of the hand can be considered as a “safe” measurement point for the upper sub-structures (from the wrist to the shoulder). On the other hand, for closer parts, as the palm skin and the fingers, it has been demonstrated that effective vibration transmission on these parts is generally over 50 Hz (Welcome et al., 2015; Dong et al., 2008). This frequency range is within the working range of the implemented damping wrist (>16 Hz), so an effective vibration reduction for these sub-structures can be hypothesized, as shown in Figure 7 for the user’s hand.

In this work, the arm direction (*x*-direction in Figure 2) was considered the most affected in terms of vibration propagation, in particular during the hammering condition. The efficacy of the robotic system in this direction can be easily appreciated by the decreasing power of the frequency spectrum in drilling SIPOREX (with hammer ON) in different configurations, as shown in Figure 7. Additionally to the high power reduction of the low frequencies (compare Figure 7C with Figures 7D,E), an important vibration suppression is also present at the hammering frequency (6400 bpm, around 107 Hz), provided by the *damped* configuration (see Figure 7E). However, with the hammer OFF, main vibrations are generated by the tool’s rotation perpendicularly to this direction, as shown in Figure 7E. In particular, two main peaks can be observed at 23 and 45 Hz, respectively. Based on the drill datasheet, the first can be associated with the maximum rotation speed, 1,400 rpm (around 23 Hz). The second results from the double cutting edge of the drill bit, which may provide a vibration at the double rotation frequency (around 45 Hz). Similar peaks can be observed in drilling SIPOREX as well, but at different frequencies (around 7 Hz and 14 Hz) since a different rotation velocity (400 rpm) was set for this. A slight decrease for these frequencies can be observed among the three different configurations in drilling wood (compare Figure 7F with Figures 7G,H). This may be due to the presence of four dampers in parallel, which provides a certain level of damping also in these directions. Nevertheless, further improvements will be oriented to a different design for better counteracting also these vibrations. The current damping wrist, for example, can be substituted with a rotary damper, and an additional damping element can be added to the user’s handle. In this way, a stronger multi-directional vibration suppression can be provided. Another option may be represented by a fully actuated arm integrated with an online adaptive damping unit. These two parts, if properly controlled and coordinated, may reject vibrations on specific frequency ranges. This system can also provide more self-interaction and collaboration between the user and the robotic device for the possibility of completely controlling arm position and orientation. However, this architecture will introduce other kinds of issues (e.g., encumbrance, weights, the need for energy sources, and control complexity) and will need further optimization to respect the functional requirements discussed in Sections 1 and 2. Nevertheless, the possibility of implementing these alternative solutions on our system will be investigated in future works.

Another interesting outcome of this experimental analysis regards the execution time. It resulted in the same duration for all the tested configurations (*without/rigid/damped*), as reported in Table 1. This is an important point in terms of productivity and work efficiency since it guarantees that the robotic system does not introduce additional delays to the task execution. Future experiments will be oriented to investigate this point in relation to the drilling precision and quality.

Another potential advantage of the proposed system is the load redistribution capacity. Preliminary results on this aspect were reported by Ciullo et al. (2018). Based on the hardware design, we can ensure a significant load reduction not only on shoulder and elbow joints but also on other weaker and more vulnerable joints such as fingers and wrist. Different from most of the upper body exoskeletons (Toxiri et al., 2015; Martinez et al., 2008), with the presented SRL, the grasping action is mostly performed by the robotic hands. This solution can reduce or eliminate fingers and wrist joint overloading, which are anatomically weaker and smaller than the torso and legs (where the load is re-distributed thanks to the SRL). Such load reduction has also been demonstrated for deltoid and biceps muscles (Huysamen et al., 2018; Rashedi et al., 2014). However, the authors also highlighted undesirable effects on the antagonist muscles and low back, which may not be negligible. These can be due to the introduction of additional masses distributed far from the user’s center of mass (the robotic arm + hand), which induce additional forces/torques. According to the literature (Kim and Nussbaum, 2019; Huysamen et al., 2018; Rashedi et al., 2014), for a comprehensive investigation of advantages/disadvantages of using this kind of systems, three main conditions shall be addressed: 1) conducting the experiments in a more realistic environment, 2) collecting data of longer exposure, and 3) involving experienced workers for the experimental validations. Future works will be oriented in this direction to collect more data and define a more accurate pros/cons ratio.

## 8 Conclusion

In this work, a supernumerary robotic arm-hand system, based on a wearable commercial gravity compensator (Armor Man), a synergy-driven under-actuated robotic hand (SoftHand), and a custom damping wrist, was presented. The system was specifically designed to improve industrial worker ergonomics in two main aspects: minimizing the load on the worker’s arm joints and reducing the vibration transmission on their arms. The first was carried out thanks to the mechanical design of the gravity compensator combined with the robotic hand. In this way, an object can be directly grasped by the robotic hand and the load is translated from small joints (as fingers and wrist) to bigger and stronger ones (e.g., shoulder, torso, and legs). The custom damping wrist design also significantly reduced vibrations when using vibrating tools, which otherwise may cause a *hand-arm vibration syndrome*. Experimental results for drilling two different materials (SIPOREX and wood) reported a vibration transmission reduction from 40 to 60% with respect to the traditional hand drilling.

## Data Availability

The raw data supporting the conclusions of this article will be made available by the authors without undue reservation.

## Footnotes

1 Official Journal of the European Communities, Directive 2002/44/EC of the European Parliament and of the Council of 25 June, 2002.
